# Hereditary and clinical insights into paraganglioma and pheochromocytoma

**DOI:** 10.1530/EO-24-0029

**Published:** 2024-10-29

**Authors:** Caitlin B Mauer Hall, Elise M Watson, Tanushree Prasad, Chandler L Myers, Jacqueline A Mersch

**Affiliations:** 1 Department of Health Care Sciences, University of Texas Southwestern Medical Center, Dallas, Texas, USA; 2 Cancer Genetics, University of Texas Southwestern Medical Center, Dallas, Texas, USA; 3 O’Donnell School of Public Health, University of Texas Southwestern Medical Center, Dallas, Texas, USA; 4 Department of Maternal-Fetal Medicine, Novant Health, Wilmington, North Carolina, USA

**Keywords:** hereditary predisposition, incidental findings, paraganglioma, pheochromocytoma, SDHx

## Abstract

**Background:**

Approximately 30–40% of paragangliomas (PGLs) and pheochromocytomas (PCCs) harbor an underlying hereditary cause. Early identification of at-risk individuals is imperative given the early onset, aggressiveness of tumors, and other tumor/cancer risks associated with hereditary PGLs/PCCs. This study analyzes the clinical presentations and genetic histories of patients with PGL/PCC and/or hereditary risk to contribute to the expanding knowledge in this rare population.

**Methods:**

A retrospective chart review identified two cohorts of patients seen in cancer genetics clinics at an academic medical center and a safety-net hospital between August 2016 and December 2022. Cohort 1 consisted of patients with likely pathogenic variants (LPVs)/pathogenic variants (PVs) in hereditary PGL/PCC predisposition genes. Cohort 2 consisted of patients with a personal history of a PGL/PCC. Demographics, personal/family history, and genetic testing outcomes were analyzed.

**Results:**

A total of 560 patients met the study criteria (Cohort 1, *n* = 364; Cohort 2, *n* = 269). In Cohort 1, 77 (21.1%) patients had an incidental LPV/PV in a PGL/PCC gene. Nearly half (*n* = 36, 46.8%) were in *SDHx* genes, with a majority in *SDHA* (*n* = 21). In Cohort 2, 86 patients tested positive for 87 LPV/PV in a hereditary cancer predisposition gene. The *SDHx* genes were most likely to have an LPV/PV identified (*SDHB n* = 24, *SDHD n* = 23).

**Conclusions:**

Multigene panels identify patients at risk for hereditary PGL/PCC, many of whom are incidentally found. While *SDHA* LPV/PVs were the most frequent incidental finding, they were less common in patients with PGL/PCC, indicating the need for longitudinal studies to better understand the prevalence and penetrance of these tumors.

## Introduction

Paragangliomas (PGLs) and pheochromocytomas (PCCs) are rare neuroendocrine tumors, with approximately 500–1600 of these tumors diagnosed per year in the United States (Aygun & Uludag 2020). PGLs and PCCs can confer high morbidity due to catecholamine secretion and cardiovascular effects, and in turn, can lead to high mortality rates if they go undiagnosed or become metastatic (Turin 2022). Thus, early detection is imperative to improve morbidity and survival outcomes.

It is currently estimated that 30–40% of PGLs/PCCs have an underlying hereditary cause (Fishbein *et al.* 2013, Aygun & Uludag 2020). Hereditary PGLs/PCCs tend to have younger ages of onset, can be more aggressive, and can be seen with a constellation of other tumors and/or cancers (van Hulsteijn *et al.* 2012, Aygun & Uludag 2020, Turin *et al.* 2022). As such, multiple medical societies and consensus groups recommend all patients with PGLs and PCCs undergo genetic testing (Pacak *et al.* 2007, Fishbein *et al.* 2021, Horton *et al.* 2022, Lenders *et al*. 2023, NCCN Neuroendocrine and Adrenal Tumors 2024). This recommendation applies regardless of the age of tumor onset, family history, or other clinical features, given that sporadic appearing PGL/PCC within a family has an approximate 11–13% likelihood of harboring a germline mutation (Brito *et al.* 2015).

PGLs and PCCs are associated with several hereditary cancer and tumor predisposition syndromes, including multiple endocrine neoplasia type 1 (MEN1), multiple endocrine neoplasia type 2 (MEN2), von Hippel–Lindau syndrome (VHL), neurofibromatosis type 1 (NF1), hereditary leiomyomatosis and renal cell cancer (HLRCC), and hereditary paraganglioma–pheochromocytoma syndrome (HPPS). Some of these conditions can present with syndromic features, such as medullary thyroid carcinoma (MEN2), renal cancers, hemangioblastomas, endolymphatic sac tumors (VHL), or uterine leiomyomas (HLRCC).

While the risk for PGL and/or PCC is low in most of these syndromes compared to their other cancer risks, as the name suggests, PGLs/PCCs are the most common tumors in HPPS. HPPS is caused by mutations in the succinate dehydrogenase (SDHx) coding genes, which include *SDHA*, *SDHB*, *SDHC*, *SDHD*, and *SDHAF2* (Nazar *et al.* 2019). Additional genes include *TMEM127* and *MAX*, along with some newer candidate susceptibility genes: *DLST, DNMT3A, EGLN1, GOT2, KIF1B, MDH2*, *PHD1*, *PHD2*, and *SLC25A11* (Fishbein *et al.* 2021, Lenders *et al.* 2023). However, the associated risk of PGL/PCC with this latter group of genes is not yet clearly understood (Fishbein *et al.* 2021).

PGL/PCC lifetime risks, screening recommendations, and management guidelines can vary drastically based on the gene identified and, in some instances, from whom it is inherited (maternally or paternally). Additionally, each syndrome listed above confers multiple tumor risks, resulting in the need for enhanced screening to provide early detection and/or prevention to help mitigate cancer risks to the patient and their at-risk relatives. As such, multigene panel tests have now become the standard of care when offering germline genetic testing to patients within this population.

Interestingly, some patients diagnosed with PGL or PCC elect to undergo a pan-cancer hereditary panel (broader than just the PGL/PCC-related genes) and are found to have likely pathogenic/pathogenic variants (LPV/PV) in genes unrelated to their PGL/PCC. A study of over 1700 individuals with PGL/PCC found that when genetic testing was restricted to *SDHB*, *SDHC*, and *SDHD* genes, one-third of individuals with PVs were missed (Horton *et al.* 2022). Conversely, LPV/PVs in PGL/PCC predisposition genes are being incidentally identified in patients undergoing pan-cancer hereditary testing panels for reasons outside of PGL/PCC (e.g. workup for hereditary breast cancer). These patients have no knowledge of PGL/PCC in their family history, thus challenging providers to make screening and management recommendations.

In this study, we describe and analyze a large cohort of patients obtained from both an academic medical center and a safety-net hospital. The data are divided into two cohorts, with the first including patients with an LPV/PV in a PGL/PCC predisposition gene, and the second cohort consisting of patients with a personal history of PGL/PCC. Personal and family histories, along with genetic testing outcomes, provide a characterization of the presentations and genetic histories of patients with PGL/PCC, hereditary risk, and incidental findings.

## Methods

Using an internal department database, a query was performed to identify two cohorts of patients seen in the cancer genetics clinics at both an academic medical center and a safety-net hospital between August 2016 and December 2022. The study was approved by the UT Southwestern IRB, study number STU-2021-1120. Patients in Cohort 1 consisted of patients from both institutions who harbored LPV/PVs in defined hereditary PGL/PCC predisposition genes (*FH, MAX, MEN1, NF1, RET, SDHA, SDHAF2, SDHB, SDHC, SDHD, TMEM127,* and *VHL*). Patients in Cohort 2 consisted of patients from both institutions who presented to genetic counseling due to a personal history of PGL/PCC.

Using the internal database as well as the electronic medical records at both institutions, patient demographics such as age, sex, race, and tumor history were collected. Personal history, family history, indication for genetic testing, and genetic testing results were reviewed and recorded for the two cohorts. Patients were excluded if they were minors, had variants in the *FH* gene that were classified as FH-deficiency carrier mutations, had variants in the *VHL* gene associated with polycythemia, or had possible mosaic results.

Subanalyses were performed within Cohort 1, including analyses of incidental findings and unaffected or asymptomatic carriers. Incidental findings were defined as patients with no personal or family history of PGL/PCC and no syndromic features for a hereditary PGL/PCC condition (e.g. medullary thyroid carcinoma, cutaneous leiomyomas, neurofibromas). Unaffected or asymptomatic carriers were patients who tested positive for an LPV/PV in a PGL/PCC gene but who were unaffected by a tumor/cancer.

The type of genetic testing ordered and the outcomes of genetic testing were recorded for patients in Cohort 2. Patients undergoing single-site or single gene analysis are defined here as ‘single gene testing’. Patients who had multiple PGL/PCC genes analyzed on one test are defined as having a ‘targeted PGL/PCC panel’, and patients who had PGL/PCC and other hereditary cancer predisposition genes analyzed are defined as having a ‘pan-cancer panel’.

### Statistical methods

Data were described as means and standard deviation for continuous variables, and frequencies with percentages for categorical variables. Wilcoxon two-sample tests for continuous variables and chi-square or Fisher’s exact tests for categorical variables were used when comparing patient characteristics across the academic medical center and safety-net hospital clinical sites. The level of statistical significance was set to *P* value <0.05. All statistical analyses were performed using SAS 9.4 (SAS Institute Inc., Cary, NC, USA).

## Results

A total of 560 patients were identified as meeting the study criteria. This total group of patients was divided into two cohorts: Cohort 1 consisted of 364 with an LPV/PV in a PGL/PCC gene, while Cohort 2 consisted of 269 patients with a personal history of PGL/PCC. Seventy-three patients overlapped between the two cohorts.

### Cohort 1: patients with LPV/PV in PGL/PCC genes

#### Population demographics

The demographics for Cohort 1 are described in Table 1. Within this cohort, patients were predominantly female (*n* = 240, 65.9%), white (*n* = 236, 64.8%), and had a mean age of 45.6 years (±15.8 years) at the time of their genetic counseling appointment. A majority of the patients (*n* = 305, 83.8%) were seen at the academic medical center compared to 59 (16.2%) seen at the safety-net hospital.

**Table 1:** Describes the demographics for patients in Cohort 1 (patients with a likely pathogenic/pathogenic variant in a paraganglioma (PGL) or pheochromocytoma (PCC) predisposition gene).

Characteristics	Total	Academic medical center	Safety-net hospital
Total *n* (%)	364 (100)	305 (83.8)	59 (16.2)
Age at date of service, mean ± s.d.	45.6 ± 15.8	46.3 ± 16.2	41.8 ± 12.8
Gender, *n* (%)			
Male	124 (34.1)	106 (34.8)	18 (30.5)
Female	240 (65.9)	199 (65.2)	41 (69.5)
Race, *n* (%)			
White or Caucasian	236 (64.8)	226 (74.1)	10 (17.0)
Black or African American	24 (6.6)	14 (4.6)	10 (17.0)
American Indian or Alaskan native	3 (0.8)	3 (1.0)	0 (0.0)
Asian	22 (6.0)	21 (6.9)	1 (1.7)
Native Hawaiian or Pacific Islander	0 (0.0)	0 (0.0)	0 (0.0)
Hispanic or Latino	73 (20.0)	35 (11.5)	38 (64.4)
Other	6 (1.7)	6 (2.0)	0 (0.0)
PGL/PCC gene positive			
*MAX*	1 (0.3)	1 (0.3)	0 (0.0)
*RET*	53 (14.6)	49 (16.1)	4 (6.8)
*SDHA*	36 (9.9)	31 (10.2)	5 (8.5)
*SDHAF2*	2 (0.6)	2 (0.7)	0 (0.0)
*SDHB*	62 (17.0)	59 (19.3)	3 (5.0)
*SDHC*	18 (5.0)	16 (5.3)	2 (3.4)
*SDHD*	39 (10.7)	34 (11.2)	5 (8.5)
*TMEM127*	6 (1.6)	5 (1.6)	1 (1.7)
*VHL*	41 (11.3)	25 (8.2)	16 (27.1)
*EGLN1*	0 (0.0)	0 (0.0)	0 (0.0)
*FH*	48 (13.2)	40 (13.1)	8 (13.6)
*KIF1B*	0 (0.0)	0 (0.0)	0 (0.0)
*MEN1*	17 (4.7)	14 (4.6)	3 (5.1)
*NF1*	41 (11.3)	29 (9.5)	12 (20.3)
Other gene positive, *n* (%)			
Yes	18 (5.0)	14 (4.6)	4 (6.8)
No	346 (95.0)	291 (95.4)	55 (93.2)

#### Genetic test results

The SDHx genes alone represented 43.1% (*n* = 157) of the total positives, with 90.4% (*n* = 142) identified at the academic medical center and 9.6% (*n* = 15) identified at the safety-net hospital. Overall, LPV/PVs were found most frequently in the *SDHB* gene (*n* = 62, 17.0%), the *RET* gene (*n* = 53, 14.6%), and the *FH* gene (*n* = 48, *n* = 13.2%). When analyzed by clinic, the gene distribution between clinics was significantly different (*P* = 0.0017). This gene distribution frequency mirrored the overall cohort at the academic medical center; however, at the safety-net hospital, LPV/PVs were most frequently found in the *VHL* (*n* = 16, 27.1%), *NF1* (*n* = 12, 20.3%), and *FH* genes (*n* = 8, 13.6%).

Eighteen (5.0%) of the 364 patients that tested positive for a gene associated with hereditary PGL/PCC also tested positive for a second hereditary cancer LPV/PV. Heterozygous *MUTYH* LPV/PV was found most frequently (*n* = 4) followed by *BRCA2* (*n* = 3).

#### Patient presentation

Of the 364 patients, 82 (22.5%) had a personal history of a PGL and/or PCC, 107 (29.4%) had syndromic features but no PGL nor PCC, 48 (13.2%) were diagnosed with a tumor/cancer not associated with a hereditary PGL/PCC condition, and 127 (34.9%) were unaffected by cancer. The difference in tumor distribution was statistically significant (*P* = 0.0002) between the patients seen at the academic medical center and the safety-net hospital (Table 2).

**Table 2:** Describes the personal and family histories of patients with likely pathogenic/pathogenic variants in paraganglioma (PGL) and pheochromocytoma (PCC) predisposition genes seen at the academic medical center and the safety-net hospital.

	Total	Academic medical center	Safety-net hospital
Personal history			
PGL and/or PCC	82	73	9
Syndromic features but not PGL/PCC	107	73	34
Different type of cancer/tumor	48	41	7
Unaffected	127	118	9
Family history			
PGL	52	48	4
PCC	16	15	1
PGL and PCC	6	6	0
Known familial mutation	130	122	8

With regard to family history, 52 (14%) patients within the total cohort had a family history of PGL only (Table 2). Sixteen (4.4%) patients within the total cohort had a family history of PCC only. Six (1.6%) patients had a family history of both PGL and PCC. Of the 364 patients, 130 (35.7%) had a known familial mutation in a PGL/PCC gene. Across all categories, the majority (>90%) of these patients were seen at the academic medical center.

#### Subanalysis 1: incidental findings

Of the 364 patients in Cohort 1, 77 (21.1%) had an incidental LPV/PV in a PGL/PCC gene. Overall, incidental findings were more frequently identified in patients at the academic medical center clinics (*n* = 63, 81.8%). However, when assessed proportionately to the volume of patients at each clinical site, patients at the safety-net hospital had a higher rate of incidental findings (safety-net hospital: *n* = 14/58, 23.7% vs academic medical center: *n* = 63/305, 20.7%).

Nearly half (*n* = 36, 46.8%) of all incidental findings were in *SDHx* genes. The most common genes with incidental findings were *SDHA* (*n* = 21, 27.3%), followed by *NF1* (*n* = 15, 19.5%). These genes were observed most frequently as incidental findings across both clinical sites.

Of the 77 patients with incidental findings, 56 (72.7%) underwent genetic testing because they met the National Comprehensive Cancer Network® (NCCN®) guidelines for Genetic/Familial High-Risk Assessment: Breast, Ovarian, and Pancreatic testing criteria at the time of their genetic counseling visit. Interestingly, 16 of the 21 (76.2%) patients with *SDHA* LPV/PVs were tested because they met these criteria. Six (28.6%) of the 21 *SDHA* patients also met the NCCN® Genetic/Familial High-Risk Assessment: Colorectal testing criteria at the time of their genetic counseling visit. Overall, seven total patients met multiple genetic testing criteria, none of which was PGL/PCC focused. Two of the 77 patients pursued genetic testing due to cascade testing for an LPV/PV in a non-PGL/PCC gene, and because they underwent pan-cancer panel testing, were identified to have an *SDHA* and an *NF1* LPV/PV, respectively.

The personal history of cancer/tumors for patients with incidental findings was analyzed at the time of genetic testing (Table 3). Four of the 77 (5.2%) patients presented with more than one cancer diagnosis. Thirty-seven (48.1%) patients were unaffected, while 24 (31.2%) presented with a personal history of breast cancer. Evaluation of all patients with an incidental *SDHx* gene (*n* = 36) showed that 16 (44.4%) were unaffected, 10 (27.8%) had a personal history of breast cancer, and three (8.3%) had a personal history of prostate cancer. An evaluation of those patients with incidental LPV/PV in *SDHA* (*n* = 21) showed that 10 (47.6%) were unaffected at the time of genetic testing, three (14.3%) had a personal history of prostate cancer, and two (9.5%) had a personal history of breast cancer. Of the 15 patients with *NF1* incidental LPV/PV, seven (46.7%) had a personal history of breast cancer.

**Table 3:** Describes the prevalence of likely pathogenic/pathogenic variants in hereditary paraganglioma/pheochromocytoma (PGL/PCC) genes in patients without a personal history of PGL/PCC.

Cancer/tumor type	Total	*FH*	*MEN1*	*NF1*	*RET*	*SDHA*	*SDHB*	*SDHC*	*SDHD*	*TMEM127*	*VHL*
Appendiceal carcinoid	1	0	0	0	0	1	0	0	0	0	0
Breast cancer	24	2	2	7	1	2	5	0	3	2	0
Colorectal cancer	2	0	0	1	0	1	0	0	0	0	0
Desmoid tumor	1	0	0	0	0	1	0	0	0	0	0
Endometrial cancer	2	0	0	0	0	2	0	0	0	0	0
Gastric cancer	1	0	0	0	0	1	0	0	0	0	0
Skin cancer (melanoma & non-melanoma)	2	0	0	1	0	1	0	0	0	0	0
Non-Hodgkin's lymphoma	1	0	0	1	0	0	0	0	0	0	0
Ovarian cancer	2	0	0	1	0	0	0	1	0	0	0
Pancreatic cancer	2	0	0	0	0	1	0	0	0	0	1
Prostate cancer	5	1	0	0	1	3	0	0	0	0	0
Urothelial cancer	1	0	0	0	1	0	0	0	0	0	0
No personal history of cancer	37	7	2	5	2	10	2	2	2	3	2

#### Subanalysis 2: unaffected or asymptomatic carriers

Of the 364 patients in Cohort 1 who were identified to have LPV/PVs in PGL/PCC genes, 43 (11.8%) were identified as unaffected or asymptomatic carriers. Forty-two of the 43 (97.7%) unaffected or asymptomatic carriers were identified at the academic medical center. Additionally, 41 of the 43 (95.3%) had a known familial mutation in a PGL/PCC gene at the time of their genetic counseling appointment. The majority (*n* = 41, 95.3%) of the LPV/PV found were in the *SDHx* genes, with LPV/PV in *SDHB* being the most frequent (*n* = 25, 58.1%).

### Cohort 2 analysis: PGL/PCC tumors

#### Population demographics

The demographics for Cohort 2 (269 patients with a personal history of PGL/PCC) are described in Table 4. Within this cohort, patients were predominantly female (*n* = 178, 66.2%), white (*n* = 184, 68.4%), and had a mean age of 53.7 years (± 15.4 years) at the time of their genetic counseling appointment. The mean age of their first PGL/PCC diagnosis was 49.4 (±17.0 years). A majority of our patients were seen at the academic medical center (*n* = 235, 87.4%) compared to 34 (12.6%) seen at the safety-net hospital.

**Table 4:** Outlines the demographics for patients in Cohort 2 (patients with a personal history of paraganglioma (PGL) or pheochromocytoma (PCC).

	Total	Academic medical center	Safety-net hospital
Total, *n* (%)	269	235 (87.4)	34 (12.6)
Age at date of service, mean ± s.d.	53.7 ± 15.4	53.8 ± 15.6	52.7 ± 14.1
Age at first PGL/PCC, mean ± s.d.	49.4 ± 17.0	49.1 ± 17.4	51.4 ± 13.7
Gender, *n* (%)			
Female	178 (66.2)	155 (87.1)	23 (12.9)
Male	91 (33.8)	80 (87.9)	11 (12.1)
Race, *n* (%)			
White or Caucasian	184 (68.4)	174 (94.6)	10 (5.4)
Black or African American	48 (17.8)	36 (75.0)	12 (25)
American Indian or Alaskan native	2 (0.7)	2 (100)	0 (0)
Asian	11 (4.1)	11 (100)	0 (0)
Native Hawaiian or Pacific Islander	1 (0.4)	1 (100)	0 (0)
Hispanic or Latino	19 (7.1)	8 (42.1)	11 (57.9)
Other	4 (1.5)	3 (75)	1 (25)
Tumor presentation, *n* (%)			
Single PGL	153 (56.9)	136 (88.9)	17 (11.1)
Multiple PGLs	27 (10.0)	24 (88.9)	3 (11.1)
Single PCC	82 (30.5)	68 (82.9)	14 (17.1)
Multiple PCCs	5 (1.9)	5 (100)	0 (0)
PGL and PCC	2 (0.7)	2 (100)	0 (0)
Family history of PGL^a^, *n* (%)			
Yes	26 (9.7)	24 (92.3)	2 (7.8)
No	243 (90.3)	211 (86.8)	32 (13.2)
Family history of PCC^b^, *n* (%)			
Yes	12 (4.5)	9 (75.0)	3 (25.0)
No	257 (95.5)	226 (87.9)	31 (12.1)
Known familial mutation, *n* (%)			
Yes	10 (3.7)	10 (100)	0 (0)
No	259 (96.3)	225 (86.9)	34 (13.1)

^a^PGL, paraganglioma; ^b^PCC, pheochromocytoma.

#### Genetic test results

Of the 269 patients, 251 (93.3%) pursued genetic testing; 122 (48.6%) elected a pan-cancer panel, 119 (47.4%) elected a targeted PGL/PCC panel, and 10 (4.0%) elected single gene testing. Ten patients presented to genetic counseling with known familial mutations; seven elected single gene testing, one elected a targeted PGL/PCC panel, and one elected a pan-cancer panel. Fifteen patients canceled genetic testing prior to receiving results.

Overall, 86 (36.4%) of the 236 patients who received genetic testing results tested positive for an LPV/PV in a hereditary cancer syndrome gene; one patient tested positive for two LPV/PVs (*SDHB* and *CHEK2*), resulting in a total of 87 LPV/PVs identified (Table 5). The average age of the first PGL or PCC in this group of patients was 40.4 years (±16.1 years). This was significantly different from the patients who tested negative for an LPV/PV in a hereditary cancer gene (average age onset of first PGL/PCC = 54.1 years, ±15.9 years, *P* < 0.0001). The *SDHx* genes were the most likely genes to have an LPV/PV identified, with the most frequent being in *SDHB* (*n* = 24) followed by *SDHD* (*n* = 23). LPVs/PVs in *SDHA* were only identified seven times.

**Table 5:** Demonstrates the paraganglioma (PGL) and pheochromocytoma (PCC) presentation of patients who were identified to harbor a likely pathogenic/pathogenic variant in any cancer predisposition gene.

Gene LPV/PV identified in	Single PGL^a^	Multiple PGLs^a^	Single PCC^b^	Multiple PCCs^b^	PGL^a^ and PCC	Grand total
*BRCA1*	1	0	0	0	0	1
*CDKN2A*	1	0	0	0	0	1
*CHEK2*	2	1	2	0	0	55
*MAX*	0	0	1	0	0	1
*MUTYH*	0	0	2	0	0	2
*NF1*	0	0	1	0	0	1
*RET*	0	0	6	2	0	8
*SDHA*	5	0	2	0	0	7
*SDHAF2*	2	0	0	0	0	2
*SDHB*	17	4	2	0	1	24
*SDHC*	6	2	0	0	0	8
*SDHD*	10	13	0	0	0	23
*SPINK1*	1	0	0	0	0	1
*VHL*	0	0	1	1	0	2
*WRN*	0	0	1	0	0	1
**Grand total**	**45**	**20**	**18**	**3**	**1**	**87** ^c^

^a^PGL, paraganglioma; ^b^PCC, pheochromocytoma; ^c^Eighty-seven mutations were identified in 86 patients; one patient had both an *SDHB* and *CHEK2* likely pathogenic/pathogenic variant.

#### Patient presentation

At the time of their genetic counseling consultation, 180 (66.9%) of the patients in Cohort 2 presented with a PGL, 27 (15.0%) of whom presented with multiple PGLs. After excluding patients who did not order genetic testing (*n* = 12) or canceled their test (*n* = 9), 159 of these patients with PGL(s) received genetic testing results. Forty-five of the 135 (33.3%) patients presenting with a single PGL tested positive for an LPV/PV in a hereditary cancer gene (Table 6). These LPV/PVs were found in PGL/PCC genes 40 (88.9%) times, most frequently in *SDHB* (*n* = 17) and *SDHD* (*n* = 10). Of the 27 patients who presented with multiple PGLs at the time of their genetic counseling consultation, 24 completed genetic testing and 19 (79.2%) tested positive for an LPV/PV in a hereditary cancer gene, all of which were in PGL/PCC genes (*SDHD* = 13, *SDHB* = 4, *SDHC* = 2).

**Table 6:** Demonstrates the genetic testing outcomes and positive rates for patients presenting with single or multiple paraganglioma(s) (PGL) or pheochromocytoma(s) (PCC).

	Total	No test	Cancelled	Resulted	LPV/PV	Positive rate (%)
PGL^a^	153	10	8	135	45	33.3
Multiple PGL^a^	27	2	1	24	19	79.2
PCC^b^	82	6	6	70	18	25.7
Multiple PCC^b^	5	0	0	5	3	60.0
PGL^a^ & PCC^b^	2	0	0	2	1	50.0
Total	269	18	15	236	86	36.4

^a^PGL, paraganglioma; ^b^PCC, pheochromocytoma.

Of the 87 (32.3%) patients who presented with PCC, five presented with multiple PCCs. After excluding patients who did not order genetic testing (*n* = 6) or canceled their test (*n* = 6), 75 of these patients with PCC(s) received genetic testing results. Eighty-two patients presented with a single PCC, 70 completed genetic testing, and 18 (25.7%) tested positive for an LPV/PV in a hereditary cancer gene (Table 6). Thirteen of the positives were specifically in PGL/PCC genes (*RET* = 6, *SDHA* = 2, *SDHB* = 2, *MAX* = 1, *NF1* = 1, *VHL* = 1). All five patients with multiple PCCs completed genetic testing, and 3 (60.0%) tested positive for an LPV/PV in a hereditary cancer gene, all of which were hereditary PGL/PCC genes (*RET* = 2,* VHL* = 1). Two (0.8%) patients presented with both a PGL and a PCC, one of whom tested positive for an LPV/PV in a hereditary cancer gene, which happened to be *SDHB*.

## Discussion

This study describes a large dataset of patients (*n* = 560) with either a hereditary predisposition to PGL/PCC or a personal history of PGL/PCC. It also encompasses a diverse set of patients from both an academic medical center and a safety-net hospital, highlighting the similarities and differences in the identification and presentation of these patients.

Our cohort of patients presenting with a personal history of PGL/PCC mirrors much of the cohorts in existing literature (Fishbein *et al.* 2013, Aygun & Uludag 2020). For example, the overall positive rate among patients with PGL/PCC was 36.4%, aligning with prior literature showing a positive rate of 20–40%. Similarly, the average age of onset of first PGL/PCC was 40.4 years (±16.1 years), which was significantly different in the population of patients with an LPV/PV in a PGL/PCC predisposition gene compared to those who tested negative for an LPV/PV. Interestingly, the positive rate in patients with multiple PGLs and/or PCC was much higher (range: 50.0–79.2%), and all LPV/PV were identified in PGL/PCC predisposition genes. This data supports the current recommendations that all patients presenting with a PGL/PCC should be offered germline genetic testing, especially those presenting with multiple tumors.

While patients with LPV/PV in PGL/PCC genes were identified in both the academic medical center and safety-net hospital settings, the distribution of genes harboring the LPV/PV differed significantly based on the clinic site. LPV/PVs were found more often in SDHx genes at the academic medical center, whereas LPV/PVs were found more frequently in syndromic PGL/PCC genes (e.g. *VHL*, *NF1*, *FH*) at the safety-net hospital. Several reasons for the difference in genotypic distribution can be speculated. First, syndromic features tend to be more recognizable, variable, and prevalent than PGL/PCCs alone. Thus, providers may be more likely to recognize, inquire about, or diagnose syndromic features, prompting a referral to genetics. This is supported by our data, as fewer patients at the safety-net hospital were seen due to a personal history of PGL/PCC, family history of PGL/PCC, or the presence of a known familial mutation. These patterns suggest the importance of educating providers on collecting family history information, understanding referral indications, and providing patient education regarding the importance of genetic testing.

Another possible explanation for the distribution of genotypes between the safety-net hospital and academic medical center may be the racial and ethnic differences between the two clinic sites. Prior studies have found that minority groups perceive genetic information as more important for cancer screening, detection, and treatment compared to non-Hispanic white individuals (Hong *et al.* 2024). Additionally, having more limited health literacy was associated with more frequent communication with providers about family health history (Kaphingst *et al.* 2016). These findings suggest that patient awareness and perception of cancer risk may not be significant contributing factors in minority patients presenting for cancer genetic counseling/testing. However, prior studies did not focus their analysis on patients with a personal or family history of PGL/PCCs. We hypothesize that navigating minority patients with a personal or family history of PGL/PCC may need to account for additional challenges other cancer populations may not face. As PGL/PCCs are rare tumors, knowledge of tumor terminology alone likely requires higher health literacy, in addition to diagnoses that may be masked by the downstream symptoms these tumors cause (e.g. tinnitus/deafness, hypertension, stroke). Furthermore, since not all PGL/PCCs are malignant, patients may not recognize the importance of sharing this information with their healthcare providers or family members, resulting in fewer referrals for family history. Further investigation is needed to better understand the exact nature behind these differences in patient populations.

It is notable that the study timeframe encompassed a period of substantial developments in genetic testing technology and genetic testing strategies. In the early years of this study, it was not uncommon for genetic testing for hereditary PGL/PCC to begin with single-gene analysis and then reflex to other genes upon receipt of negative results. Additionally, the discovery of additional PGL/PCC predisposition genes also evolved during the study timeframe. As next-generation sequencing was developed and the genes available for analysis broadened, it became more common to identify incidental LPV/PV, especially in PGL/PCC predisposition genes. While almost half of the patients with PGL/PCCs in this study had pan-cancer testing, the other half had PGL/PCC-targeted testing. The reverse thought process can also be applied to patients undergoing pan-cancer panels for non-PGL/PCC indications; as multigene and pan-cancer panels evolved, PGL/PCC predisposition genes were added over time. Therefore, it is unknown how many more incidental results may have been identified in this cohort if pan-cancer testing had been performed for all patients, and thus incidentals are likely underreported in this study.

Our findings also highlight the utility and challenges of pan-cancer panel testing. Approximately 20% of our patients with LPV/PVs in a PGL/PCC gene were identified incidentally. This means that one in five individuals in our population with a PGL/PCC predisposition would have gone undetected if non-PGL/PCC targeted panel testing had been performed. This data emphasizes how pan-cancer panel testing identifies patients at risk for tumors/cancers that may not be expected based on personal/family history, and how it allows patients the potentially life-saving opportunity for additional surveillance/management. However, it also emphasizes the need for a better understanding of the prevalence of LPV/PV in these genes within the general population, as well as the penetrance of tumor risk.

Furthermore, when assessing patients with LPV/PVs in PGL/PCC genes, we found that 5% have a second LPV/PV in a non-PGL/PCC gene. This is consistent with literature describing the rate of individuals with more than one LPV/PV (Agaoglu *et al.* 2024) and further highlights the benefit of pan-cancer panel testing in identifying at-risk individuals that may not have been detected with smaller, more targeted panels. While heterozygous *MUTYH* LPV/PV was the most frequent second finding in this study and is less likely to impact surveillance/management recommendations, nearly 40% of patients with a second LPV/PV had a second LPV/PV that would impact clinical recommendations.

Almost half of the patients with incidental LPV/PVs in PGL/PCC genes were unaffected at the time of genetic counseling/testing. Comparably, when analyzing our cohort of patients with incidental findings in *SDHA*, which comprised nearly 30% incidental findings, half of the patients were again unaffected. This highlights the importance of pre-test genetic counseling regarding the possibility of incidental findings, especially in an era of pan-cancer testing. Additionally, as LPV/PVs in *SDHA* were seen far less in our population of patients diagnosed with PGL/PCC, this provides an example of the need to better understand the penetrance and frequency information for all PGL/PCC genes. The current approach of ‘one-size-fits-all’ management for patients in this population poses a dilemma that calls into question unnecessary screenings, impacts healthcare dollar spend, and potentially induces anxiety for patients. Large-scale and longitudinal studies are needed to better elucidate genotype-phenotype correlations in this population. Until then, a multidisciplinary approach, including the involvement of a genetic counselor, is necessary to facilitate a shared decision-making management plan with the patient. Similar commentary provided by Skefos *et al.* exists regarding LPV/PVs in *SDHA* specifically and also highlights the impact genetic information has on cascade testing (Skefos *et al.* 2024). As the expansion in the use of pan-cancer panel testing raises the challenge of how to approach surveillance and management for patients that do not fit the expected clinical presentation, additional research in these populations will better characterize how to care for patients with inherited PGL/PCC risks.

## Conclusion

In the era of multigene panel testing, more patients are being identified with hereditary risks for PGL/PCC. Patients presenting with PGL/PCC demonstrate a positive rate of 30%, with younger individuals having a higher likelihood of an LPV/PV, supporting existing data. However, with broad, pan-cancer panels being offered routinely, many incidental LPV/PV findings occur in PGL/PCC genes, indicating the need for longitudinal studies to better characterize the prevalence and penetrance of these tumors across a diverse patient population.

## Declaration of interest

The authors declare that there is no conflict of interest that could be perceived as prejudicing the impartiality of the study reported.

## Funding

This work was supported in part by the Texas Society of Genetic Counselors (Fall 2022 Grant Award).

## Ethics

The study was approved by the UT Southwestern IRB, study number STU-2021-1120.

## Author contribution statement

CBMH conceived the study, collected and assembled data, performed data analysis and interpretation and contributed to writing the manuscript, and is accountable for all aspects of the work. EMW collected and assembled data, performed data analysis and interpretation and contributed to writing the manuscript, and is accountable for all aspects of the work. TP performed data analysis and interpretation and contributed to writing the manuscript. CLM collected and assembled data and contributed to writing the manuscript. JAM conceived the study, collected and assembled data, performed data analysis and interpretation and contributed to writing the manuscript, and is accountable for all aspects of the work.
